# Is dignity therapy feasible to enhance the end of life experience for people with motor neurone disease and their family carers?

**DOI:** 10.1186/1472-684X-11-18

**Published:** 2012-09-20

**Authors:** Brenda Bentley, Samar M Aoun, Moira O’Connor, Lauren J Breen, Harvey Max Chochinov

**Affiliations:** 1 Western Australian Centre for Cancer and Palliative Care, Curtin Health Innovation Research Institute, Curtin University, GPO Box U1987, Perth, WA, 6845, Australia; 2 School of Psychology and Speech Pathology, Curtin Health Innovation Research Institute, Curtin University, Perth, Australia; 3 Manitoba Palliative Care Research Unit, Department of Psychiatry, University of Manitoba, Winnipeg, Canada

**Keywords:** Motor neurone disease, Amyotrophic lateral sclerosis, Palliative care, Existential distress, Family carers, Dignity therapy

## Abstract

**Background:**

Development of interventions that address psychosocial and existential distress in people with motor neurone disease (MND) or that alleviate caregiver burden in MND family carers have often been suggested in the research literature. Dignity therapy, which was developed to reduce psychosocial and existential distress at the end of life, has been shown to benefit people dying of cancer and their families. These results may not be transferable to people with MND. The objectives of this study are to assess the feasibility, acceptability and potential effectiveness of dignity therapy to enhance the end of life experience for people with motor neurone disease and their family carers.

**Methods/design:**

This is a cross-sectional study utilizing a single treatment group and a pre/post test design. The study population will comprise fifty people diagnosed with MND and their nominated family carers. Primarily quantitative outcomes will be gathered through measures assessed at baseline and at approximately one week after the intervention. Outcomes for participants include hopefulness, spirituality and dignity. Outcomes for family carers include perceived caregiver burden, hopefulness and anxiety/depression. Feedback and satisfaction with the intervention will be gathered through a questionnaire.

**Discussion:**

This detailed research will explore if dignity therapy has the potential to enhance the end of life experience for people with MND and their family carers, and fill a gap for professionals who are called on to address the spiritual, existential and psychosocial needs of their MND patients and families.

**Trial registration:**

ACTRN Trial Number: ACTRN12611000410954

## Background

As a result of elevated interest in hastened death at the end of life by people with motor neurone disease (MND), also known as amyotrophic lateral sclerosis (ALS), numerous studies have examined factors that affect quality of life, psychological health, and end-of-life distress in this population. Findings indicate that quality of life in MND-diagnosed individuals is independent of physical decline [1,2]; that interest in hastened death is correlated with hopelessness [3,4]; and that MND patients with higher levels of spirituality and sense of meaning experience less end of life distress [5,6]. This research has resulted in a call to develop psychosocial interventions for use with the MND population that will bolster hopefulness, spirituality, and meaning [7,8]; however, very little work has been done to develop and implement such interventions.

MND is a family disease, and family carers carry an exceptional burden by providing a high level of care, often for the duration of the illness. Family carers of people with MND are more depressed than people with MND overall [9]. As time goes on and dependency increases, family carers exhibit increasing levels of distress symptoms [10,11]. Studies on the quality of life in MND family carers suggests that perceived caregiver burden can be alleviated by finding positive meaning [12,13] and by supporting a sense of hope [14].

Dignity therapy, a brief psychotherapeutic intervention based on empirical research into the concept of dignity at the end of life [15], has proven successful at increasing hope, sense of meaning and will to live in a palliative care population, where most patients had cancer diagnoses. Dignity therapy offers people with terminal illness the opportunity to create a generativity document. In a recorded interview guided by a counsellor or health care professional, the participant is invited to recount aspects of their life they want remembered, find meaning and purpose to their life, and express final words or advice. The interview is transcribed and edited, and a final dignity therapy transcript is returned to the participant to share with others as they wish.

A pilot study of dignity therapy produced positive results for participants and family members. A heightened sense of dignity was reported in 76% of participants, an increased sense of purpose was reported by 68%, an increased sense of meaning was reported by 67%, and 47% reported an increased will to live [16]. A recent randomised controlled trial reported similar outcomes [17]. In the pilot study, family members were also positive about the intervention, with 95% reporting they would recommend dignity therapy, 78% reporting that it helped them during their time of grief, and 77% believing the document would be a continuing source of comfort [18].

Nonetheless, because these studies both utilized a primarily cancer population, the results are not transferable to people with MND. Ability to communicate, cognitive acuity, stage of illness, baseline levels of distress and demographic features are some of the factors that may vary in this population and make implementation of dignity therapy difficult. The delivery of the intervention may require modification, i.e. to be performed at an earlier stage in the disease process or by utilizing assisted communication methods. Therefore, feasibility testing of dignity therapy with the MND population is warranted [19,20].

Moreover, while the previous dignity therapy study focused on the intervention’s positive influence on the bereavement experience of family members, it did not look at how the intervention may affect the carer during the caring experience.

### Aims and objectives

The aims of this study are to assess the feasibility, acceptability and potential effectiveness of dignity therapy to enhance the end of life experience for people with MND and their family carers. The specific objectives are to:

a) Determine whether dignity therapy is likely to increase hope, meaning and dignity in people with MND.

b) Determine whether dignity therapy is likely to increase hope, and decrease anxiety, depression, and perceived burden in family carers of people with MND.

c) Determine whether dignity therapy is acceptable to people with MND and their family carers.

d) Determine whether it is feasible to provide dignity therapy to people with MND.

e) Pilot methods for a future randomized controlled trial.

## Methods

### Study design

This is a cross-sectional study utilizing a single treatment group and a repeated measures pre/post test design. A control group is not being utilized due to 1) the small MND population available in Western Australia 2) access issues to people with MND, and 3) the fact that dignity therapy (nor any other psychosocial intervention) has yet to be tried with this palliative care population and the feasibility of dignity therapy needs to be tested before proceeding to an RCT. The study design has been modelled to reduce bias and increase validity where possible. For example, the short duration of the intervention coupled with utilizing a single post-testing point one week after the intervention will minimize confounding variables.

### Ethical approval

This study has been approved by the Curtin University Human Research Ethics Committee (19/2011).

### Participants

The sample will comprise 50 adults diagnosed with MND who are registered with the MND Association of Western Australia. A second group will consist of up to 50 family carers of the participants.

#### People with MND

##### Inclusion criteria

Persons with a diagnosis of MND aged 18 and over who are able to communicate in English are included in the study. As this is a feasibility study, participants who are unable to communicate verbally may participate if they are able to utilize an assisted communication method. Communication issues will be explored and reported in the findings. There is no selection criteria based on stage of the disease as MND is a fatal disease with no hope of remission making an end-of-life intervention appropriate at any time after diagnosis. Participants will not be screened for existential or psychosocial distress; however, these will be assessed at baseline.

##### Exclusion criteria

Participants who are unable to provide informed consent, either due to cognitive issues or illness severity will be excluded. Cognitive impairment will be screened using the ALS-Cognitive Behavioral Screen [36]. Â If the participant receives a score of less than ten, the participant and family carer will be excluded from the study.

#### Family carers

A family carer of each participant will be invited to take part in the study, comprising a second research population of up to 50 family carers. A family carer is defined as the person indicated by the participant as the primary family carer. The family carer must be at least 18 years of age, able to provide informed consent and able to communicate in English. If the family carer does not wish to participate, then the person with MND remains eligible to take part in the study.

### The intervention and study procedures

The intervention will be administered by the researcher, a counselling psychologist undertaking a PhD by research at Curtin University in Western Australia. The researcher has been trained in dignity therapy at an intensive workshop by Professor Harvey Chochinov, who developed the intervention and who performed the empirical studies upon which the intervention is based.

Recruitment will be undertaken through the MND Association of Western Australia (MNDAWA). MNDAWA will liaise directly with participants to protect the identity of MND-diagnosed individuals. Letters will be sent out to all members of MNDAWA and potential participants are identified when they telephone the researcher or return a copy of the letter expressing their interest. The researcher will first speak with the person with MND or family carer to elaborate further on the nature of the study and answer any questions. If the participant wishes to proceed, a meeting will be scheduled. If time permits, information sheets and consent forms will be mailed in advance of the meeting to provide time to consider the study and discuss their participation with family and health care providers. All meetings will occur at a time of the participant’s choosing and in their care environment. Since the participant’s condition can fluctuate, the timing of the contacts can be flexible and meetings rescheduled. The researcher will make detailed notes of these experiences in order to include information about timing issues in the findings.

At the initial meeting, the researcher will review the participant and family carer information and consent forms with the participant/family carer dyad, check for understanding and answer any outstanding questions. Written consent will be obtained and MND participants will be tested for cognitive impairment that is significant enough to exclude them from the study. If the participant and family carer remain in the study, socio-demographic and health questionnaires and baseline measures will be collected from each. An appointment for the first dignity therapy session will be made, ideally within two to three days. The researcher will provide the participant a copy of the dignity therapy question framework so that they may begin reflecting on their responses.

Dignity therapy question protocol

Tell me a little about your life history; particularly the parts that you either remember most or think are the most important? When did you feel most alive?

Are there specific things that you would want your family to know about you, and are there particular things you would want them to remember?

What are the most important roles you have played in life (family roles, vocational roles, community service roles, etc.)? Why were they so important to you, and what do you think you accomplished in those roles?

What are your most important accomplishments, and what do you feel most proud of?

Are there particular things you feel still need to be said to your loved ones or things that you would want to take time to say once again?

What are your hopes and dreams for your loved ones?

What have you learned about life that you would want to pass along to others? What advice or words of guidance would you wish to pass along to your son, daughter, husband, wife, parents, other(s)?

Are there words or perhaps even instructions that you would like to offer your family to help prepare them for the future?

In creating this permanent record are there other thing you would like included?

[16] at p. 5522.

The next meeting is the primary dignity therapy session, where participants are invited to address and record themes, thoughts and feelings about themselves and their lives that they would like remembered. The question framework is flexible, and provides a guide for the researcher to shape the interview by following the participant’s cues. If the participant desires, a family carer may be present during the interview to provide emotional support and help facilitate the interview. If participants require a second or third interview session due to communication issues, fatigue, or the breadth of information they would like to share, these will be scheduled as soon as possible.

After recording has finished, a verbatim transcript will be prepared (usually within 24–48 hours). The researcher will shape the interview into a narrative using the learned dignity therapy editing method, which omits non-starters and irrelevant sections (such as interruptions) and tags content that needs to be clarified or perhaps omitted due to the harm it could inflict on recipients. Another appointment will be made with the participant as soon as practicable to read through and edit the transcript. In this session, the participant will be invited to make corrections, clarifications or additions as desired. In the final dignity therapy session, the final bound transcript is given to the participant. The participants may have as many copies of the document as they wish and share them with whoever they choose.

**Figure 1:**
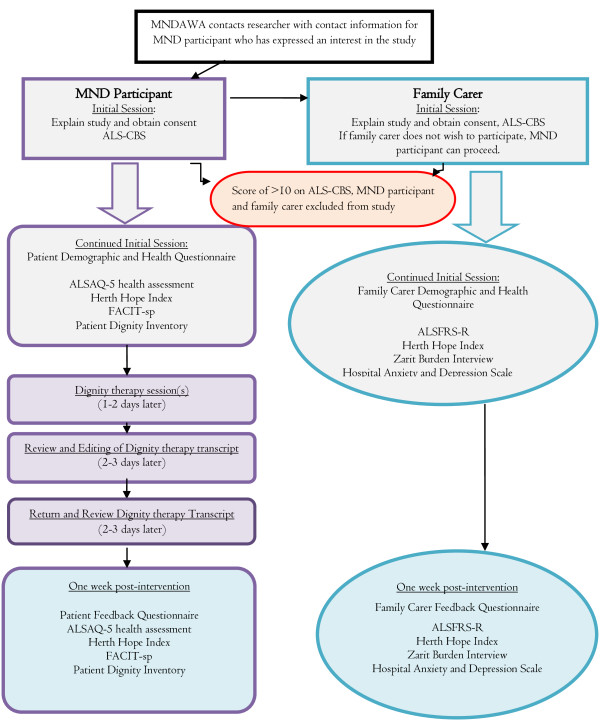
Study Design Flow Chart.

Post-testing will occur with both the participant and family carer one week after the final dignity therapy document has been returned. To reduce response bias, post-testing questionnaires will be sent out and returned via mail. A research officer (not related to the project) from the Western Australian Centre for Cancer and Palliative care will perform the post-testing if assistance is needed to complete the questionnaires. A single point of post-testing soon after completion of the sessions is planned in order to reduce moderating variables. Further, the possible immediate impact of the intervention is being examined for the purposes of this feasibility study and not the longevity of the outcomes, which can be assessed in future studies. A study design flow chart is shown in Figure 1.

### Measures and outcomes

The measures chosen have been validated and are quick and easy to administer and use, in order to decrease the burden on the study population. Brief versions of the outcome measures have been selected where available.

#### Primary outcome measure for people with MND

The primary outcome measure is the participant’s sense of hopefulness. This will be assessed utilizing the Herth Hope Index [21,22], a validated instrument developed for use with the terminally ill. Distressed MND patients score higher on hopelessness than distressed cancer patients at the end of life [23], and interest in hastened death or suicidal ideation in the MND population has been shown to correlate with hopelessness [3,24], which indicates that the level of hopefulness a key factor of psychological distress in this disease population.

#### Secondary outcome measures for people with MND

Secondary outcome measures used to assess potential effectiveness include, 1) dignity, which will be measured with the Patient Dignity Inventory (PDI) [25], a validated measure which evolved directly from the empirical studies into dignity concerns in the terminally ill, and; 2) spiritual well-being, which will be measured with the Functional Assessment of Chronic Illness Therapy-Spiritual Well-Being Scale (FACIT-sp-12) [26], a validated measure showing strong internal reliability. To assess the acceptability of dignity therapy, a modified version of the dignity therapy Patient Feedback Questionnaire [17] is being utilized to collect information on the views, experiences and opinions of the participants about the intervention. To assess the feasibility of the intervention, data will be collected about the time taken to organize and conduct the dignity therapy sessions, any special accommodations made in the delivery of the intervention, deviations from the dignity therapy protocol, and reasons for non-completion. The researcher will also record, through the use of a journal, observations and experiences of delivering the intervention, including positive and negative participant responses.

#### Primary outcome measure for family carers

The primary outcome is the family carer’s sense of perceived burden, which will be measured through the Zarit Burden Inventory [27]. Research of caregiver burden in MND family carers has documented the considerable burden attached to caring for persons with MND [28,29]. Caregiver burden increases as patient function declines [30-32]. Studies indicate that MND caregiver burden can be alleviated by finding positive meaning [12,13] and by supporting a sense of hope [14]. It can be inferred that dignity therapy may impact hope and meaning, thereby alleviating caregiver burden. In the alternative, whether this intervention increases burden is a factor that also must be considered when evaluating its overall impact.

#### Secondary outcome measures for family carers

Secondary outcome measures used to assess the potential impact of dignity therapy on family carers includes 1) hopefulness, utilizing the Herth Hope Index [21]. The HHI has been used successfully in studies with family caregivers of terminally ill patients [33], and 2) anxiety and depression, which will be measured with the Hospital Anxiety and Depression Scale [34], an instrument often used with family caregivers showing strong reliability and validity. To assess the acceptability of dignity therapy to family carers, a modified version of the dignity therapy Family Feedback Questionnaire [17] is being utilized to collect information on the views, experiences and opinions of the family carers about the intervention.

#### Demographic and health status

Demographic and health status information collected from persons with MND will include disease specific health-related quality of life utilizing the Amyotrophic Lateral Sclerosis Assessment Questionnaire-5 [35], cognitive behavioural functioning from the carer’s perspective utilizing the ALS Cognitive Behavioral Screen [36], age, gender, and health history.

Demographic and health status information collected from family carers will include the level of disability of the care recipient utilizing the Amyotrophic Lateral Sclerosis Functional Rating Scale-R [37,38], cognitive behavioural functioning from the carer’s perspective utilizing the ALS Cognitive Behavioral Screen [36], age, gender, relationship to the person with MND, caring hours per day, employment status, and health history.

### Analysis

For summarizing purposes, descriptive statistics will be obtained for demographic variables. To assess the possible impact of the intervention on the psychosocial and existential concerns of the participant and family carer, pre and post intervention comparisons for each outcome variable will be carried out using Wilcoxon’s signed rank-sum tests, given that the main outcome variable measured will not be normally distributed [16]. Spearman’s rank correlation coefficient will be calculated to assess the possible correlation between variables of interest. It is anticipated that there will be a post-intervention improvement on all psychosocial measures for both the participant and family carer after the dignity therapy intervention. Open-ended responses in feedback questionnaires will be coded and analysed using descriptive statistics. The IBM SPSS version 20 statistical software package will be used for all analyses. A p value less than 0.05 is considered to be statistically significant.

## Discussion

Several studies, as well as MND practice guidelines, suggest the need to develop and utilize interventions that will support hopefulness, a sense of meaning, and dignity in order to alleviate psychosocial and existential distress in persons with MND [7,8]. Despite this, very little has been done to develop or implement such interventions. In fact, an extensive literature search completed for this study revealed there were no psychosocial interventions specifically designed or tailored to alleviate existential distress and improve the quality of life of persons with MND. This research will begin to fill this gap, providing a possible solution to a concern about this specific population, as well as continue to advance the overall focus of alleviating psychosocial distress at the end of life, an area of palliative care which has been widely acknowledged as being in need of improvement.

This study will determine if dignity therapy is likely to be effective in enhancing the end of life experience for both people with MND and their family carers, if it is acceptable to people with MND and their family carers, and if it is feasible to offer the intervention to this population. It will explore what allowances or modifications might need to be made to deliver the intervention to people who are sometimes unable to communicate verbally. It will provide a preliminary examination of how cognitive or neurobehavioral issues encountered in persons with MND might affect the intervention, including the completion and sharing of a generativity document that reflects a true sense of the person with MND. Finally, if the intervention is unsuccessful, this study will determine what factors contributed to a negative outcome, as well as any unexpected consequences to persons with MND or their families.

This research has implications for psychological and health care professionals who work with people with motor neurone disease and other neurological disorders, as well as those who work in palliative care settings. Both are often called on to address the spiritual, existential and psychosocial needs in their patients. If dignity therapy proves to be effective, it could be a relatively brief and easy to administer intervention that could be made available to people with MND. This study has the potential to provide a precise intervention to ameliorate psychosocial and existential distress, as well as improve the quality of care provided to people with MND and their family carers.

## Competing interests

The authors declare they have no competing interests.

## Authors’ contributions

SA and HC conceived the study. BB, SA, MO and HC designed the study utilizing methods developed by HC in previous dignity therapy pilot and RCT studies. BB drafted the article and will carry out the research. SA, MO, LB and HC will supervise the research. HC developed dignity therapy. All authors made substantial contributions to the critical revision of the article and approved the final content.

## Pre-publication history

The pre-publication history for this paper can be accessed here:

http://www.biomedcentral.com/1472-684X/11/18/prepub
